# Energy Aware Swarm Optimization with Intercluster Search for Wireless Sensor Network

**DOI:** 10.1155/2015/395256

**Published:** 2015-03-30

**Authors:** Shanmugasundaram Thilagavathi, Bhavani Gnanasambandan Geetha

**Affiliations:** ^1^Department of Information Technology, Institute of Road and Transport Technology, Erode, Tamilnadu 638 316, India; ^2^Department of Computer Science and Engineering, K. S. Rangasamy College of Technology, Tiruchengode, Tamilnadu 637 215, India

## Abstract

Wireless sensor networks (WSNs) are emerging as a low cost popular solution for many real-world challenges. The low cost ensures deployment of large sensor arrays to perform military and civilian tasks. Generally, WSNs are power constrained due to their unique deployment method which makes replacement of battery source difficult. Challenges in WSN include a well-organized communication platform for the network with negligible power utilization. In this work, an improved binary particle swarm optimization (PSO) algorithm with modified connected dominating set (CDS) based on residual energy is proposed for discovery of optimal number of clusters and cluster head (CH). Simulations show that the proposed BPSO-T and BPSO-EADS perform better than LEACH- and PSO-based system in terms of energy savings and QOS.

## 1. Introduction

A sensor network deploys many sensor nodes inside a phenomenon or close to it. The position of the sensor nodes can be randomly deployed and does not need predetermined locations which ensure easy deployment in inaccessible terrain. Sensor networks comprise different sensors including seismic, magnetic, visual, thermal, acoustic, infrared, and radar sensors which monitor various ambient conditions. Sensor nodes can be designed to monitor continuously and are useful in event detection, location sensing, and actuators control. Due to its versatility wireless sensor network (WSN) promises applications in many areas [1].

**Figure 1:**
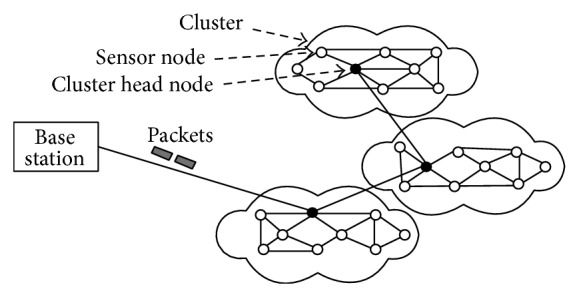
Wireless sensor network.

Sensor networks data aggregation is challenging due to its characteristic of limited energy, processing power, and transmission range [2, 3]. Global addressing for deploying large number of sensors is difficult with currently available technology and hence conventional IP-based protocols are inapplicable to sensor networks. Contrary to communication networks, most sensor networks applications need sensed data flow from multiple regions to a specific link and generate data traffic that contains redundancy as many sensors generate similar data in a phenomenon's proximity. This should be exploited by routing protocols to improve energy/bandwidth use.  Figure 1 shows the architecture of a simple WSN organized hierarchically with the data being aggregated by the cluster head (CH) from its members and then routed to the base station (BS). CH can be special nodes or elected at every round depending on the quality of service (QoS) criteria.

Multihop routing is an important service needed for WSN. Internet and mobile Ad hoc network (MANET) routing techniques fail to perform well in WSN. Internet routing assumes reliable wired connections and hence there are infrequent packet errors, but in WSN this is not the case. Similarly, MANET routing protocols assume symmetric links between two connections which is not the case with WSN as most of the traffic is unidirectional [4].

WSN routing can be classified based on network structure as flat, hierarchical, or location-based. Further, such protocols are classified as multipath-based, negotiation-based, query-based, coherent-based, and QoS-based depending on protocol operation. All nodes play similar roles in flat networks and energy saving is achieved by using efficient sleep techniques in MAC protocol. Hierarchical protocols form clusters with a designated CH to aggregate and transfer data to the BS. These techniques normally depend on TDMA time slots to sleep and achieve energy efficiency. Location-based protocols use position information to relay data to desired regions and not the entire network [5]. Query-based data-centric protocols depend on desired data naming to eliminate redundant transmissions [6].

Load balancing has been effectively used to allocate traffic amongst different paths to avoid forming congested areas and at the same time allow the energy consumed to be distributed among the entire network [7, 8]. For energy efficient WSN routing, the main objective would be the energy constraints within the network. Being a NP complete problem, an ideal route can be efficiently found based on heuristic or metaheuristic techniques [9]. Maintaining WSN routes is nontrivial as energy restrictions and node status changes result in frequent/unpredictable topological changes.

Low energy adaptive clustering hierarchy (LEACH) is a popular protocol in WSN. LEACH is a clustering protocol in which the random rotations of local cluster heads are utilized in order to distribute energy load among all sensor nodes in the network. LEACH is popular since it provides scalable network by limiting the communication present inside different clusters; single-hop routing is possible from sensor node to cluster head which results in saving of energy in the network and it increases the network lifetime. The sensor nodes that are not CH communicate based on the schedule created by the CH when using time division multiple access (TDMA) protocol. LEACH operation can be organized into two phases: setup phase and a transmission phase [10]. In the setup phase, the nodes are classified into clusters with one CH in each cluster. In transmission phase, cluster heads collect data from the nodes present in those clusters and transfer the processed information to the BS. In LEACH, CHs are rotated every round to improve the overall network life time compared to fixed CH-based algorithms [11]. LEACH uses a random CH selection technique [12] where in each sensor it selects a random number between 0 and 1. If the selected number is less than predetermined threshold values then the node becomes a CH. The following equation shows the threshold computation in LEACH:

(1) $$\begin{array}{c} t=\left\{\begin{array}{cc} \frac{P}{1-P\times \left[r\mathrm{mod}1/p\right]}, & \text{if}\;\;n\in G,\\ 0, & \text{otherwise}, \end{array}\right. \end{array}$$

where *P* is the probability that a node will become the CH, *r* is the current round, and *G* is the set of nodes that are yet to become CH. The major role of CH is to aggregate data from all its members and to send the data packets to BS. Though performance of LEACH in terms of energy savings is good, it suffers from many drawbacks such as random selection of CH and not considering energy consumption. LEACH is neither suitable for large area nor suitable for densely deployed network with nonuniform distribution of CHs. Various enhancements for LEACH have been proposed in literature to improve the energy savings. Kumar et al. [13] did an extensive survey on clustering algorithms based on LEACH reported in WSN literature. Singh et al. [14] compared various clustering-based algorithm techniques including LEACH, LEACH-C, and PEGASIS.

Cluster formation being NP complete and various techniques to find optimal solution using evolutionary and swarm intelligence algorithm have been proposed in literature. Particle swarm optimization (PSO), a popular swarm intelligence algorithm, has been extensively used for solving optimization due to its simple concept and low computational cost. For clustering algorithm, PSO has been applied in many different ways [9, 15–18]. Wang et al. [15] made a survey on PSO-based solution for WSN issues. Sobe et al. [16] proposed WSN-based algorithm single cluster head PSO (SPSO) and double cluster head PSO (DPSO) over varied sensor network areas. Results show that SPSO performs better in network life extension in small network area and for large network area DPSO performs better by load balancing. Latiff et al. [17] proposed an energy-balanced unequal clustering (EBUC) protocol with PSO algorithm. EBUC adopts energy aware multihop routing to reduce cluster heads energy consumption for intercluster communication which results in increased network lifetime. Kulkarni and Venayagamoorthy[9] proposed an adaptive mutation probability binary particle swarm optimization (AMPBPSO) algorithm to search for the best placement scheme to ensure network reliability and cost reduction. Jiang et al. [18] proposed WSN performance analysis using artificial neural networks (ANNs). PSO is used as learning algorithm to find an optimized path and to ensure an energy efficient network. In recent times Kuila and Jana [19] investigated PSO for energy efficient clustering and routing using a multiobjective function which improves the overall quality of service. Other swarm intelligence (SI) algorithms which have been successfully investigated for WSN network life time improvement include artificial bee colony (ABC) [20, 21], Cuckoo search [22], and ant colony optimization [23, 24]. A good SI algorithm shows improvements in global search and fast convergence for the globally best solution. To further improve the global search various hybrid algorithms have been proposed in literature to improve the existing SI algorithms.

Over thirty modifications of particle swarm optimization have been proposed in literature [25–31] which have shown improvements over PSO proposed by Kennedy and Eberhart [32] in various domains. To the best of our knowledge not many investigations have been carried out in the area of WSN using modified PSO. This work investigates the impact of hybridization of PSO and its applicability in WSN.  Section 2 defines the problem statement, Section 3 gives the detailed methodology of this work, Section 4 explains the results and discusses the same, and Section 5 concludes this paper.

## 2. Problem Statement

One of the main objectives in WSN design is increasing the network life time. Cluster-based schemes improve network life; however, most popular algorithms including LEACH use the concept of one hop for intracluster and intercluster communication which leads to larger average transmission distance. In this work PSO-based cluster formation technique with multiple objectives is proposed for intracluster data aggregation with a connected dominated set- (CDS-) based intercluster communication based on energy objective. The multiobjective function in this work considers both energy and packet delivery ratio (PDR) which are normalized in the objective function.

WSN can be represented by a connected unidirectional graph represented by *G* = (*N*, *L*) where *N* represents the vertices and consists of (*n*_1_, *n*_2_,…, *n*_*i*_) nodes. *L* represents the edges between the nodes given by (*l*_1,2_, *l*_1,3_,…, *l*_*i*,*j*_). Since the objective is to improve the QoS of the network, each edge is defined by the QoS optimization attributes and can be formulated as

(2) $$\begin{array}{c} l_{i,j}=\overset{n}{\underset{k=1}{\sum }}\alpha_{k}w_{i,j}^{k}\;\text{such}\;\;\text{that}\;\;\overset{n}{\underset{k=1}{\sum }}\alpha_{k}=1. \end{array}$$

Since *l*_*i*,*j*_ is constrained by the transmission range of the node, the connectivity between node *i* and *j* can be represented by

(3) $$\begin{array}{c} l_{i,j}=\left\{\begin{array}{cc} 1, & \text{if}\;\text{nodes}\;i\;\text{and}\;j\;\text{are}\;\text{within}\\ & \text{communication}\;\text{distance}\\ 0, & \text{otherwise}. \end{array}\right., \end{array}$$

The objective is to minimize

(4) $$\begin{array}{c} \min f_{i}\left(x\right)=\alpha \left(\min\left(\frac{1}{\text{PDR}}\right)\right)+\beta \left(\min\left(\frac{E_{i}^{r}}{E_{\text{initial}}}\right)\right), \end{array}$$

where *E*_*i*_^*r*^ is the remaining energy in node *i* and *E*_initial_ is the initial energy in the node.

Saravanan and Madheswaran [21] used minimum spanning tree (MST) for establishing intercluster communication. The technique proposed creates load balancing issues; even if multiple paths exist the communication occurs only through the path defined by the MST algorithm. In this work during the setup phase efficient clusters are formed with CH selection based on binary particle swarm optimization (BPSO) with an improved transfer function. Once the CH is elected, the intercluster communication among the CHs to reach the sink is established using energy aware connected dominating set (EADS) to find the optimal route from the suboptimal solutions.

## 3. Methodology

The assumptions made in the network model for simulations are as follows.

- The network is assumed to be square in nature.
- The base station is located in the centre of the network.
- Sensor nodes are placed randomly following uniform distribution.
- Each sensor node has omnidirectional antenna of uniform range.
- All nodes have uniform initial energy.
- Each member node transmits its data to the specific CH selected in that round.
- Cluster heads can transmit the data to the base station using multihops.
- Nodes and base station are stationary.

**Figure 2:**
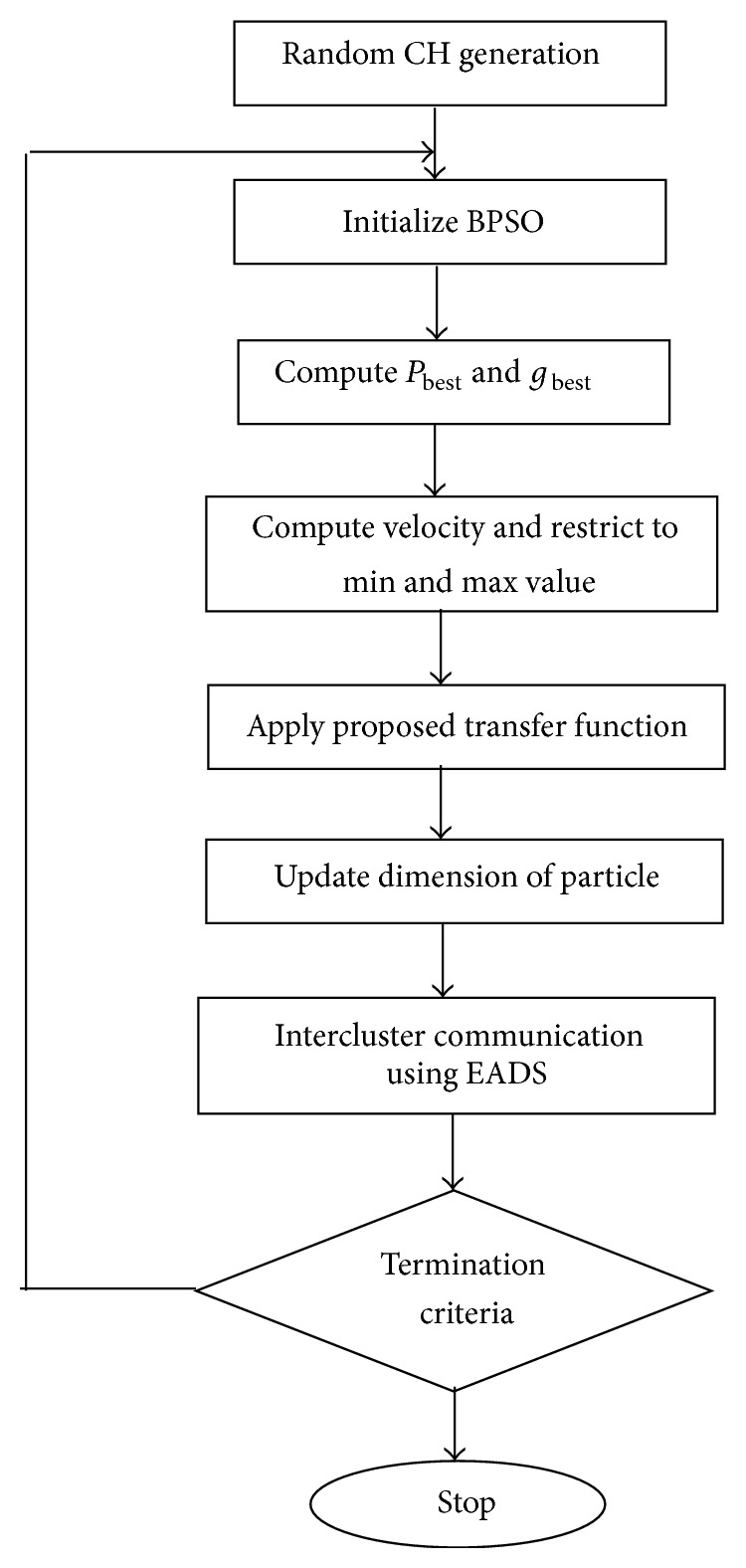
Proposed system for optimal cluster formation and CH selection.

The first order radio model proposed in [11] for one hop scenarios is adapted for multihop scenario. The flow chart of the proposed method is shown in Figure 2.

PSO was initially proposed by Kennedy and Eberhart in 1995 as a stochastic optimization technique to solve discrete and continuous problems using random variables. PSO simulates the social behaviour of insects/birds. In PSO, each candidate solution is represented as a particle or as an individual bird or as an individual fish in search space [33]. The particle moves to a better location based on its individual knowledge and the knowledge gained by the swarm to find an optimal solution using the fitness function. The initial solution is generated randomly within the boundaries of the search space [34]. PSO uses the cognition model to perform the local search while it uses its social skills to perform global search. During each iteration, the next position of the particle is computed based on its cognition and social skills [35].

Each particle consists of its own position and velocity that can be randomly initialized. After initialization, the particles search their best positions with its or neighboring experience. Every particle maintains two positions called *p*_best_ and *g*_best_. The *p*_best_ represents the particles' own best position and *g*_best_ is the global best position among all the particles. The position and velocity [36] of each particle are updated based on

(5) Vit+1=Vi(t)+α1∗r1(pbest−ci) +α2∗r2(gbest−ci),cit+1=cit+Vit+1,

where *V*_*i*_(*t*) represents the current velocity, *p*_best_ is particle's best position, *g*_best_ is the global best position among all the particles, *r*_1_ and *r*_2_ are two numbers generated randomly between 0 and 1, *α*_1_ and *α*_2_ are acceleration coefficients, and *c*_*i*_ is the current particle position.

Selection of cluster heads from eligible nodes can be seen as a discrete binary search space problem. The nodes can flip depending on whether it is selected as a CH or not. Since PSO is represented using binary strings, the velocity and position computation can be modified accordingly. The initial population is represented by nodes where c represents a node that is selected as one of the CHs for that initial random solution. For a dimension *d* in the search space, *c*_*id*_^*k*^ indicates that a node is placed for particle *i* in period *d* at iteration *k*. In other words, *c*_*id*_^*k*^ is a binary value such that *c*_*id*_^*k*^ = 1, if the node is selected as CH; else *c*_*id*_^*k*^ = 0 otherwise. During the setup phase each node can become a CH with a probability of 0.5. Specifically, if *p*(0,1) > 0.5, then *c*_*id*_^0^ = 1; else *c*_*id*_^0^ = 0. To avoid ambiguous results in this work, the velocity values are restricted to minimum and maximum values and are given by

(6) Vik=Vmin⁡,Vmax⁡=−8,8,where  Vmin⁡=−Vmax⁡.

The velocity of particle *i* in the *d*th dimensions can be established by

(7) $$\begin{array}{c} v_{id}^{0}=V_{\min}+\left(V_{\max}-V_{\min}\right)*\text{rand}\left(\right). \end{array}$$

Since binary values are used to represent the solutions, the velocities can take values as in

(8) $$\begin{array}{c} h\left(v_{id}^{k}\right)=\left\{\begin{array}{cc} V_{\max}, & \text{if}\;\;v_{id}^{k}>V_{\max},\\ v_{id}^{k}, & \text{if}\;\;\left|v_{id}^{k}\right|\leq V_{\max},\\ V_{\min}, & \text{if}\;\;v_{id}^{k}<V_{\min}. \end{array}\right. \end{array}$$

Once the current velocity is found, [37] applied the transfer function shown in (9) to update the velocity [37]:

(9) $$\begin{array}{c} \text{transfer}\left(v_{id}^{k}\right)=\frac{1}{1+e^{-v_{id}^{k}}}. \end{array}$$

Transfer function plays an important role in flipping the position. Larger velocities should have very high probability of flipping, whereas small value of velocity should have lower probability. In this work a novel transfer function is proposed and given in

(10) $$\begin{array}{c} \text{transfer}\left(v_{id}^{k}\right)=\frac{1}{\left(1+e^{\left(-\tanh\left(v_{id}^{k}\right)*2\pi \right)}\right)}. \end{array}$$

The dimensions *d* of the particle *i* are updated as

(11) $$\begin{array}{c} x_{id}^{k}=\left\{\begin{array}{cc} 1, & \text{if}\;\;p\left(0,1\right)<\text{transfer}\left(v_{id}^{k}\right),\\ 0, & \text{otherwise}. \end{array}\right. \end{array}$$

**Figure 3:**
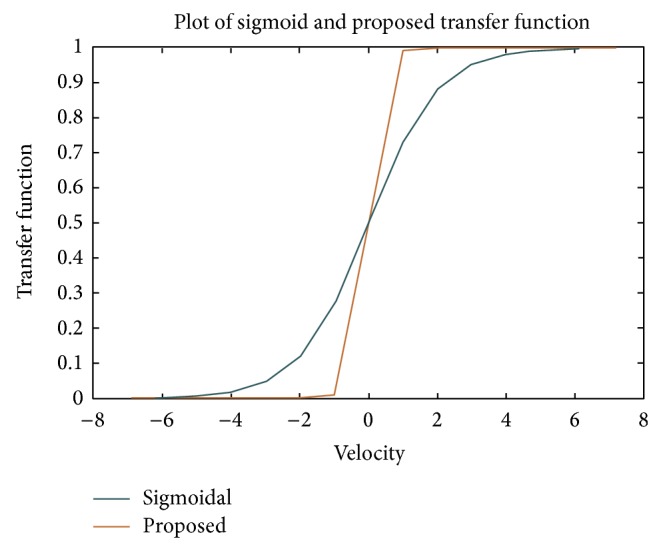
The proposed transfer function used to flip binary value.

Figure 3 shows the plot of the proposed transfer function compared to sigmoidal transfer function used in literature. The proposed transfer function shows better flipping parameters for lower velocities in a very narrow field allowing the search to converge better. Once the clusters are formed and CH is elected during the setup phase, the route to the BS from each CH is to be determined.

This work proposed an energy aware dominating set (EADS) to find optimal dominating CH to reach the destination. In graph theory dominating set (DS) is defined as a subset of vertexes such that each node in the vertex is adjacent to at least one node in subset of vertices. Connected dominating set (CDS) is a DS of graph G which creates a subgraph by adding any vertex such that independence property of the set is broken. A detailed review of DS can be found in [38]. In this work CDS is adapted to be energy aware across the edges such that the optimal features expected in the proposed EADS are as follows.

**Figure 4:**
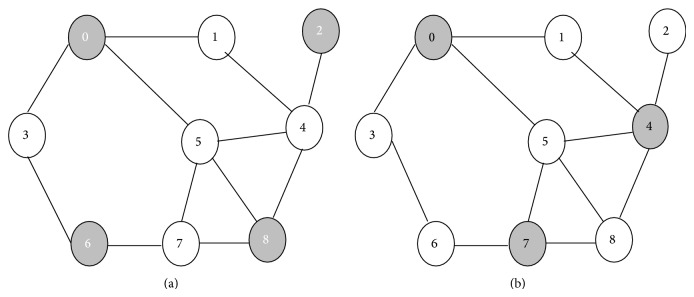
Figure 4(a) shows a DS with 4 CHs and Figure 4(b) shows a DS with 3 CHs.

(i) The DS should have minimum number of CHs.  Figure 4 shows two DSs with Figure 4(b) showing minimum number of CHs in the DS.

(ii) The total Euclidean distance between resultant DS nodes to the BS should be minimal. The shortest distance between CH and BS is the sum of the hop distance between the two. Using gamma probability density function model, the posterior distribution of sum of the hop distance for a given Euclidean distance is given by

(12) $$\begin{array}{c} P\left(D_{\text{hop}}\;|\;D_{\text{ed}}\right)=\frac{P\left(D_{\text{ed}}\;|\;D_{\text{hop}}\right)\cdot P\left(D_{\text{hop}}\right)}{\sum_{i=1}^{N}P\left(D_{\text{ed}}\;|\;i\right)\cdot P\left(i\right)}, \end{array}$$

where *D*_hop_ is the hop distance, *D*_ed_ is the Euclidean distance, *N* is the number of nodes in the DS, and *P*(*i*) is the probability that a random CH in the DS is *i* hop away from BS.

(iii) Remaining energy between the selected DS nodes should be greater than the average energy among all the nodes and is derived using

(13) $$\begin{array}{c} T_{e,{\text{ch}}_{i}}=\frac{E_{i}}{\sum_{i=1}^{N}E_{\text{remaining}}}\geq T. \end{array}$$

The proposed technique for intercluster communication uses the minimum number of CHs to reach the base station. The route is optimized by selecting nodes which are closer to the BS while ensuring that only CH with higher energy is selected.

## 4. Experimental Setup and Results

Simulations were carried out using LEACH, GA, and PSO proposed by Kennedy and Eberhart which we call PSO-K adapted to binary operation [37], PSO-SD [39], BPSO with proposed transfer function (10) (BPSO-T), and PSO-EADS with the parameters shown in Table 1.

**Figure 5:**
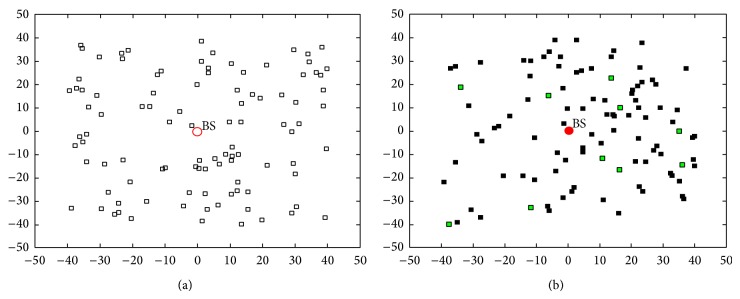
(a) An initial random placement of nodes used in our experimental setup. (b) Randomly selected CH by the initial solution of PSO.

Figure 5(a) shows the random layout of the nodes within the network area of 100 sq·m and the BS located at (0, 0). The proposed technique chooses CH randomly as shown in Figure 3(b) with the selected CH shown in green color. This solution can be seen as suboptimal as some of the randomly selected CHs are on the edge of the network. However, these become good candidate solutions for flipping during the fitness evaluation and computation of the new velocity. The proposed transfer function helps in faster convergence. The gradient at the initial iterations indicates sharp flipping characteristic of the transfer function. The convergence factor improves over 45% compared to BPSO-K which uses the classic sigmoidal transfer function. Similarly, PSO-SD showed improvements in convergence compared to PSO-K.

**Figure 6:**
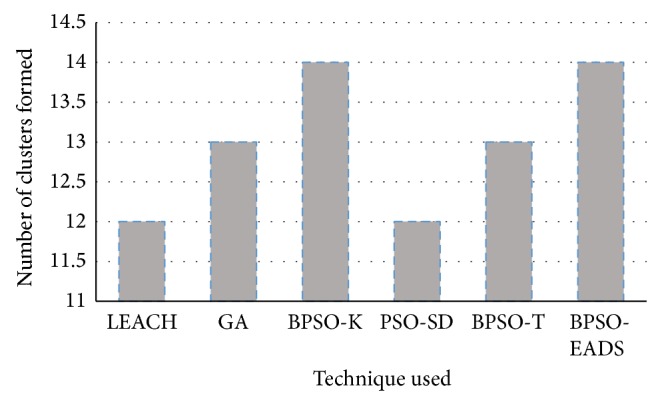
Average number of clusters formed in the first 10 rounds.

The average number of clusters formed during the initial 10 rounds is shown in Figure 6.

**Figure 7:**
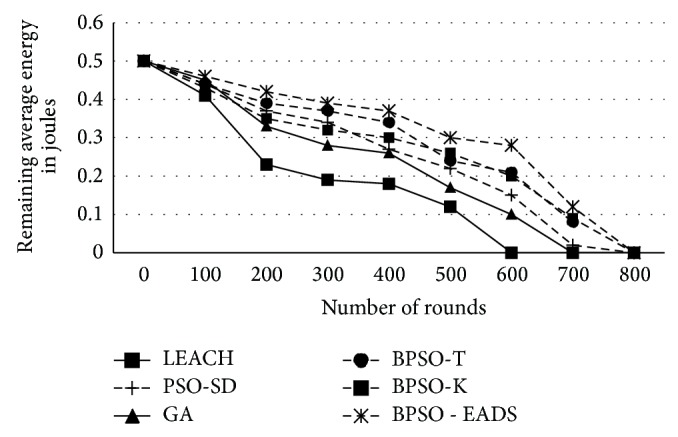
Average energy in the network after each round.

The number of clusters formed using binary particle swarm optimization is higher compared to LEACH, GA, and PSO-SD. Higher number of clusters reduces the intracluster distance among nodes and hence improves both the packet delivery ratio and energy efficiency as the transmission power used by these nodes will be lower. The increased cluster formation is utilized by EADS to form better intercluster communication. This is evident in the remaining energy in the network as the number of rounds increases as shown in Figure 7.

**Figure 8:**
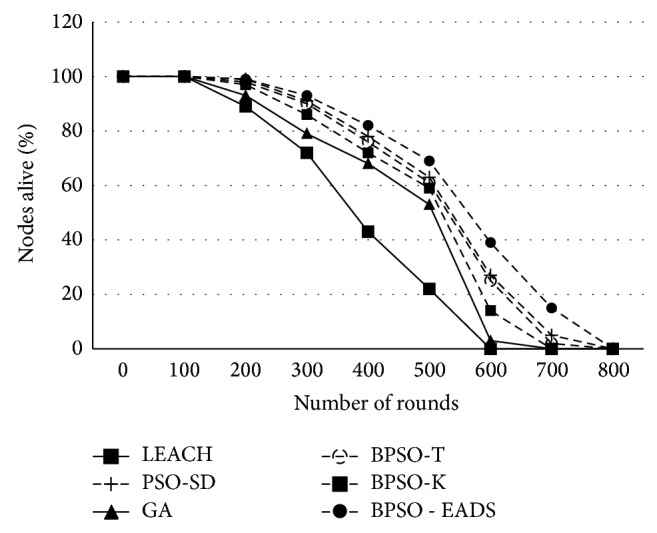
Number of nodes alive across various rounds.

Except for LEACH and GA, all techniques retain more than 50% of their energy for the first 400 rounds while GA-based cluster formation and CH selection of the retained energy during the same period are about 35% only. However, with improved intracluster communication BPSO-EADS shows better energy management for about 75% of the network life time after which it falls steeply as in other techniques. This energy management directly translates to more number of nodes being alive as seen in Figure 8. Except for LEACH, all other techniques retain 50% of the nodes for more than 550 rounds which is an improvement of over 40% compared to LEACH. In small networks, where all nodes are able to reach the BS with less than two hops, the proposed technique significantly improves the usability of the network which may not be in the case of LEACH. Due to the inherent single hop characteristic of LEACH, nodes closer to the BS die first compared to the techniques used by PSO-SD and PSO-EADS.

**Figure 9:**
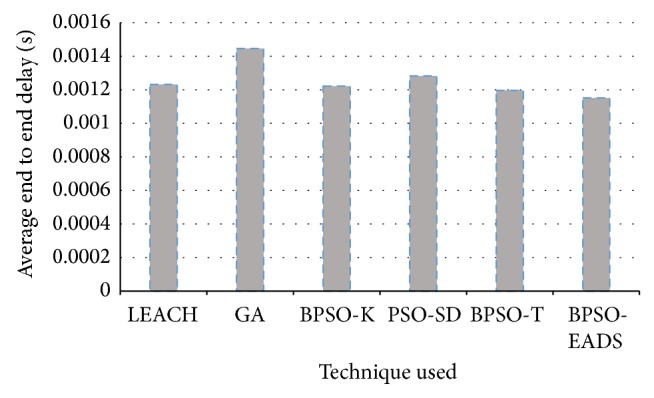
Average end to end delay over 500 rounds.

**Figure 10:**
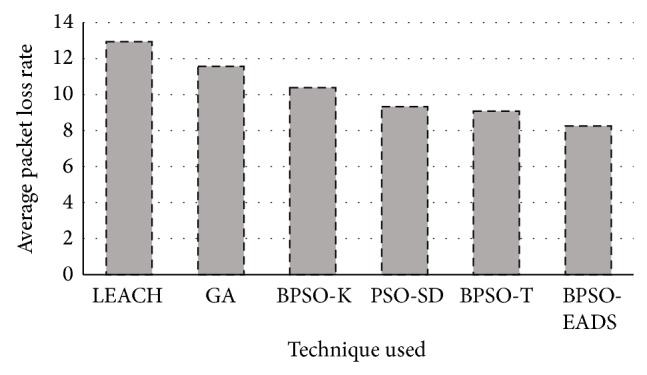
Packet loss rate over 500 rounds.

Quality of service also plays a very important role and a major requirement for current applications. The average packet loss rate and average end to end delay across 500 rounds is shown in Figures 9 and 10.

All the binary PSO methods have lower end to end delay with an average decrease of 1.6% compared to LEACH and 17.6% compared to GA. GA shows higher end to end delay compared to LEACH by 15.8%. However, this may not be significant if the data can tolerate some delay. However, the average packet loss rate is higher in LEACH compared to all other techniques as seen in Figure 10.

The packet loss rate is statistically similar between PSO-SD and PSO-T. However, the proposed intercluster communication using EADS has shown the least packet loss and is lower than LEACH by 44.19% and lower than BPSO-SD by 12.18%. Compared to classic PSO, PSO-EADS reduces the packet loss rate by 22.86%. BPSO-EADS has shown overall improvement in energy savings and QoS parameters of the network. It is also seen that PSO-based techniques perform better than GA-based technique where two-point crossover with uniform mutation was used in the experimental setup.

## 5. Conclusion

In this work, particle swarm optimization was revisited and improvements were investigated to optimize the clustering problem in wireless sensor network. The objective was to improve the energy efficiency of the network and improve the overall quality of service. In the proposed binary particle swarm optimization algorithm, an improved transfer function was investigated along with intercluster communication using a modified connected dominating set technique using the energy criteria of the cluster heads. Simulation showed that the proposed technique improved over LEACH and existing PSO algorithms in both QoS and energy savings.

## Figures and Tables

**Table 1:** Simulation parameters.

Sensor nodes taken	100 nodes
Transmission power of node	0.005 w
Initial energy of nodes	0.52 J
Amplification coefficient of the free space model	10 pJ-/b
Amplification coefficient of the multipath transmission model	0.0025 pJ-/b
Circuit loss	50 nJ/b
Data packet length	8000 b
Control packet length	120 b
*α*_1_ acceleration coefficient	0.5
*α*_2_ acceleration coefficient	0.5
*r*_1_	Random number between 0 and 1
*r*_2_	Random number between 0 and 1
Initial number of particles	20

## References

[1] Akyildiz I. F., Su W., Sankarasubramaniam Y., Cayirci E. (2002). Wireless sensor networks: a survey. *Computer Networks*.

[2] Karlof C., Wagner D. (2003). Secure routing in wireless sensor networks: attacks and countermeasures. *Ad Hoc Networks*.

[3] Hill J. L. (2003). *System architecture for wireless sensor networks [Ph.D. thesis]*.

[4] Frey H., Rührup S., Stojmenović I. (2009). Routing in wireless sensor networks. *Guide to Wireless Sensor Networks*.

[5] Al-Karaki J. N., Kamal A. E. (2004). Routing techniques in wireless sensor networks: a survey. *IEEE Wireless Communications*.

[6] Akkaya K., Younis M. (2005). A survey on routing protocols for wireless sensor networks. *Ad Hoc Networks*.

[7] Tang J., Xue G., Zhang W. Interference-aware topology control and QoS routing in multi-channel wireless mesh networks.

[8] Yang Y., Wang J., Kravets R. (2005). *Interference-Aware Load Balancing for Multihop Wireless Networks*.

[9] Kulkarni R. V., Venayagamoorthy G. K. (2011). Particle swarm optimization in wireless-sensor networks: a brief survey. *IEEE Transactions on Systems, Man and Cybernetics Part C: Applications and Reviews*.

[10] Heinzelman W. B., Chandrakasan A. P., Balakrishnan H. (2002). An application-specific protocol architecture for wireless microsensor networks. *IEEE Transactions on Wireless Communications*.

[11] Heinzelman W. R., Chandrakasan A., Balakrishnan H. Energy-efficient communication protocol for wireless microsensor networks.

[12] Gao T., Greenspan D., Welsh M., Juang R. R., Alm A. Vital signs monitoring and patient tracking over a wireless network.

[13] Kumar V., Jain S., Tiwari S. (2011). Energy efficient clustering algorithms in wireless sensor networks: a survey. *International Journal of Computer Science Issues*.

[14] Singh S. S., Kumar M., Saxena R., Priya (2012). Application of particle swarm optimization for energy efficient wireless sensor network: a survey. *International Journal of Engineering Science & Advanced Technology*.

[15] Wang L., Ye W., Wang H., Fu X., Fei M., Menhas M. I. (2012). Optimal node placement of industrial wireless sensor networks based on adaptive mutation probability binary particle swarm optimization algorithm. *Computer Science and Information Systems*.

[16] Sobe A., Fehérvári I., Elmenreich W. FREVO: a tool for evolving and evaluating self-organizing systems.

[17] Latiff N. M. A., Tsimenidis C. C., Sharif B. S., Ladha C. Dynamic clustering using binary multi-objective particle swarm optimization for wireless sensor networks.

[18] Jiang C.-J., Shi W.-R., Xiang M., Tang X.-L. (2010). Energy-balanced unequal clustering protocol for wireless sensor networks. *The Journal of China Universities of Posts and Telecommunications*.

[19] Kuila P., Jana P. K. (2014). Energy efficient clustering and routing algorithms for wireless sensor networks: particle swarm optimization approach. *Engineering Applications of Artificial Intelligence*.

[20] Karaboga D., Gorkemli B., Ozturk C., Karaboga N. (2014). A comprehensive survey: artificial bee colony (ABC) algorithm and applications. *Artificial Intelligence Review*.

[21] Saravanan M., Madheswaran M. (2014). A hybrid optimized weighted minimum spanning tree for the shortest intrapath selection in wireless sensor network. *Mathematical Problems in Engineering*.

[22] Yang X.-S., Deb S. (2014). Cuckoo search: recent advances and applications. *Neural Computing and Applications*.

[23] Kadri B., Feham M., Mhammed A. (2014). Efficient and secured ant routing algorithm for wireless sensor networks. *International Journal of Network Security*.

[24] Kim J.-Y., Sharma T., Kumar B., Tomar G. S., Berry K., Lee W.-H. (2014). Intercluster ant colony optimization algorithm for wireless sensor network in dense environment. *International Journal of Distributed Sensor Networks*.

[25] Hu Y., Ding Y., Hao K., Ren L., Han H. (2014). An immune orthogonal learning particle swarm optimisation algorithm for routing recovery of wireless sensor networks with mobile sink. *International Journal of Systems Science*.

[26] Gandomi A. H., Yun G. J., Yang X.-S., Talatahari S. (2013). Chaos-enhanced accelerated particle swarm optimization. *Communications in Nonlinear Science and Numerical Simulation*.

[27] Goksal F. P., Karaoglan I., Altiparmak F. (2013). A hybrid discrete particle swarm optimization for vehicle routing problem with simultaneous pickup and delivery. *Computers & Industrial Engineering*.

[28] Wang H., Sun H., Li C., Rahnamayan S., Pan J.-S. (2013). Diversity enhanced particle swarm optimization with neighborhood search. *Information Sciences*.

[29] He J., Guo H. (2013). A modified particle swarm optimization algorithm. *TELKOMNIKA Indonesian Journal of Electrical Engineering*.

[30] Xu W., Geng Z., Zhu Q., Gu X. (2013). A piecewise linear chaotic map and sequential quadratic programming based robust hybrid particle swarm optimization. *Information Sciences*.

[31] Chen W.-N., Zhang J., Lin Y. (2013). Particle swarm optimization with an aging leader and challengers. *IEEE Transactions on Evolutionary Computation*.

[32] Kennedy J., Eberhart R. Particle swarm optimization.

[33] Kennedy J. (2010). Particle swarm optimization. *Encyclopedia of Machine Learning*.

[34] Tu C. J., Chuang L. Y., Chang J. Y., Yang C. H. (2007). Feature selection using PSO-SVM. *IAENG International Journal of Computer Science*.

[35] Jabeen H., Jalil Z., Baig A. R. Opposition based initialization in particle swarm optimization (O-PSO).

[36] Imran M., Hashim R., Elaiza A. K. N., Irtaza A. (2014). Stochastic optimized relevance feedback particle swarm optimization for content based image retrieval. *The Scientific World Journal*.

[37] Taşgetiren M. F., Liang Y. C. (2004). A binary particle swarm optimization algorithm for lot sizing problem. *Journal of Economic and Social Research*.

[38] Blum J., Ding M., Thaeler A., Cheng X. (2005). Connected dominating set in sensor networks and MANETs. *Handbook of Combinatorial Optimization*.

[39] Singh B., Lobiyal D. K. (2012). A novel energy-aware cluster head selection based on particle swarm optimization for wireless sensor networks. *Human-Centric Computing and Information Sciences*.

